# Fertility counseling among family physicians in Turkey: a cross-sectional survey of knowledge, attitudes, and practices

**DOI:** 10.3389/fmed.2026.1884680

**Published:** 2026-07-10

**Authors:** Mehmet Faruk Olcenoglu, Merve Olcenoglu, Havva Demircioglu, Gamze Savci, Ayse Zehra Ozdemir Kaya

**Affiliations:** 1 Department of Gynecology and Obstetrics, Karamanoglu Mehmetbey University Faculty of Medicine, Karaman, Türkiye; 2 Department of Family Medicine, Karamanoglu Mehmetbey University Faculty of Medicine, Karaman, Türkiye; 3 Department of Gynecology and Obstetrics, VM Medikal Park Pendik Hospital, Istanbul, Türkiye

**Keywords:** family physicians, fertility preservation, health knowledge, oocyte cryopreservation, primary health care

## Abstract

**Background:**

There are limited data on how family physicians approach fertility counseling in Turkey. We aimed to assess family physicians’ knowledge, attitudes, and counseling practices regarding fertility, reproductive planning, and oocyte cryopreservation (OCP) in Turkey.

**Methods:**

An online, cross-sectional survey was conducted among family physicians practicing in family health centers or community health centers across Turkey between January and June 2026. Physicians were recruited until a predetermined sample size of at least 400 valid responses to the researcher-developed survey was reached, which included the collection of participant data and the fertility counseling questionnaire.

**Results:**

The proportion of family physicians supporting the proactive discussion of patients’ reproductive plans was 34.2%, while 53.6% supported discussing age-related fertility decline and 62.6% supported discussing OCP with appropriate patients. Correct identification of the age range at which mild and pronounced fertility declines began was achieved by 60.6 and 54.1% of participants, respectively. Self-reported familiarity with OCP was lower than the support shown for counseling about OCP. Familiarity with OCP was higher among younger and unmarried physicians. A significant portion of participants anticipated that insurance coverage and clarification of legal regulations would increase the frequency of counseling.

**Conclusion:**

In Turkey, attitudes toward fertility counseling at the primary care level do not always align with contemporary knowledge. The findings suggest a need for structured education, particularly regarding age-related fertility decline, the realistic limits of *in vitro* fertilization (IVF) success, and indications for OCP, as well as institutional and policy frameworks to facilitate counseling.

## 1 Introduction

Female fertility is lost predictably with age (1), with the decline beginning around 32 years of age and becoming more pronounced after 37 years of age (1, 2). In many societies, this biological reality has transformed from a theoretical concept into a matter of direct clinical importance due to delayed childbearing (3, 4). Fertility decline often begins years before a patient seeks treatment for infertility (1). Therefore, fertility counseling should be viewed not merely as a topic addressed only when pregnancy cannot be achieved, but as a preventive measure that should be considered while reproductive plans are still being formulated (5).

Systematic reviews show that individuals of reproductive age frequently overestimate the ages at which age-related declines in fertility begin and become pronounced (6). The problem is not limited to the general public. Gaps in knowledge regarding age thresholds and the success rates of assisted reproductive technologies have also been reported among healthcare professionals. Even among obstetricians and gynecologists, the age at which a marked decline in fertility begins may be underestimated, whereas the success of assisted reproduction may be overestimated (7, 8). The goal of preconception care is to identify and reduce biomedical, behavioral, and social risks before pregnancy (9). Family physicians maintain regular, ongoing contact with individuals of reproductive age and are therefore in a unique position to address reproductive intentions and risks that may affect fertility within routine care, making primary care the most natural clinical setting for fertility counseling (10, 11). Discussions on this topic are particularly meaningful when initiated early, because delayed counseling and referral can limit available options (12, 13).

Oocyte cryopreservation (OCP) was initially established for fertility preservation before gonadotoxic treatment but has since emerged as a fertility-planning option (14, 15). The American Society for Reproductive Medicine emphasizes the need for balanced OCP counseling because of uncertainties regarding efficacy (16, 17). Physician-focused studies indicate that support for discussing age-related fertility decline does not always align with OCP feasibility (12). In Turkey, it is unclear how family physicians approach fertility counseling. This study aimed to assess the counseling attitudes and practices of family physicians in Turkey regarding age-related fertility decline, reproductive planning, and OCP use, along with their knowledge levels and personal/professional characteristics.

## 2 Materials and methods

### 2.1 Study design and setting

This cross-sectional, self-administered survey study was conducted between January and June 2026. The target population comprised all family physicians practicing in primary healthcare centers in Turkey, which are divided into two structures: Family Health Centers (FHC) and Community Health Centers (CHC). No geographic restrictions were applied.

The study was approved by the Local Scientific Medical Research Ethics Committee of Karamanoğlu Mehmetbey University Faculty of Medicine (Approval date: 06.01.2026; decision no: 27–2026/22). All procedures were conducted in accordance with the principles of the Declaration of Helsinki. Informed consent was obtained electronically from all participants before survey commencement.

### 2.2 Participants

Physicians were enrolled through a convenience approach via online professional networks. All family physicians working as specialists or general practitioners in an FHC or CHC at the time of the study were eligible to participate, regardless of age, sex, or years of clinical practice. Physicians in other specialties, respondents who left more than 20% of survey items unanswered, and those who did not provide electronic informed consent were excluded. The required minimum sample size was estimated at 400, based on a finite-population correction formula applied to the total number of family physicians actively practicing in Turkey (*N* = 29,763; Ministry of Health, December 2025). Assuming maximum response variability (*p* = 0.50), a 95% confidence level (*z* = 1.96), and a 5% margin of error, the base sample size of 384 was adjusted to 380 after finite population correction and further inflated to 400 to account for an estimated 5% non-response rate.

### 2.3 Survey instrument

Data were collected using a structured, self-administered questionnaire developed by the research team specifically for this study. The instrument was organized into four thematic sections. Section 1 captured demographic information, including age, sex, marital status, parental status, years of clinical practice, and province of practice. Section 2 assessed attitudes towards fertility awareness counseling through three binary (Yes/No) items: whether family physicians should proactively initiate conversations about patients’ reproductive plans (Q7), whether age-related fertility decline should be addressed (Q8), and whether the latter should be incorporated into routine health visits (Q9). Respondents who indicated they do not address this topic routinely were asked to identify their reasons from a list of six non-mutually exclusive options (Q10). Section 3 contained four knowledge items: the age range at which the decline in female fertility begins (Q11); the age range at which a marked decrease begins (Q12); and the approximate live birth probability per *in vitro* fertilization (IVF) cycle in women younger (Q13) and older (Q14) than 35 years, each presented as multiple-choice questions. Section 4 assessed attitudes toward oocyte cryopreservation (OCP), including self-reported familiarity (Q15), willingness to initiate counseling with eligible patients (Q16), likelihood of discussing or referring for OCP in four clinical states rated on a 5-point scale (Q17), views on the legal permissibility of elective OCP for social indications (Q18), support for insurance reimbursement (Q19), and anticipated behavioral changes with respect to financial (Q20) or legislative (Q21) reform scenarios. Response categories considered most consistent with current evidence were defined *a priori* by the research team, with the age thresholds for fertility decline informed by existing committee opinions and the cited literature (1, 2): 30–34 years for the onset of slight decline, 35–39 years for marked decline, and approximate live birth probabilities of 30–39% per cycle for women younger than 35 years and 0–19% for women older than 35 years.

The questionnaire was drafted by the research team, comprising specialists in obstetrics and gynecology and family medicine, based on a review of the relevant literature on fertility awareness and preconception care. Draft items were reviewed for content and face validity by five independent specialists, revised accordingly, and pilot-tested with 11 family physicians not included in the analysis to assess clarity and completion time. Formal psychometric validation and reliability testing were not performed.

The survey was administered via an online data collection platform (Google Forms) and distributed through professional physician communication networks. No personal identifiers were collected, and it was made clear that participation was entirely voluntary and anonymous. The first page of the survey displayed an informed consent statement. Only respondents who electronically confirmed their consent were permitted to proceed. Of the 542 physicians who accessed the survey link and provided electronic informed consent, 401 submitted valid, sufficiently complete responses, corresponding to a completion rate of 74.0% among those who accessed the survey. Because the total number of physicians exposed to the invitation through the professional network could not be determined, a conventional response rate based on the full invited population could not be calculated.

### 2.4 Outcomes

The primary outcomes were the proportions of family physicians who endorsed family physician-initiated proactive counseling on reproductive plans (Q7), age-related fertility decline (Q8), and OCP (Q16). Secondary outcomes included self-reported familiarity with OCP (Q15), accuracy of responses to knowledge items on the timing of fertility decline (Q11, Q12) and IVF success rates (Q13, Q14), scenario-specific counseling likelihood (Q17), and attitudes toward the legalization (Q18), insurance coverage (Q19), and hypothetical behavioral changes related to financial (Q20) or legislative (Q21) reforms. Subgroup analyses examined associations between all outcomes and five demographic characteristics: age (<35 vs. ≥35 years), sex (female vs. male), marital status (married vs. not married), parental status (has children vs. no children), and duration of clinical practice (<10 vs. ≥10 years).

### 2.5 Statistical analysis

All statistical analyses were performed using IBM SPSS Statistics, version 27.0 (IBM Corp., Armonk, NY, United States). A two-tailed *p*-value of less than 0.05 was considered statistically significant. The normality of continuous variables was assessed through visual inspection of histograms and Q-Q plots. As these variables did not follow a normal distribution, they were reported as medians (25th–75th percentile). Categorical variables were reported as frequencies and percentages. Between-group comparisons for non-normally distributed continuous variables were conducted using the Mann–Whitney *U*-test. Associations between categorical variables were evaluated using the Pearson’s chi-square test, Fisher’s exact test, or the Fisher–Freeman–Halton exact test, as appropriate. For tabular presentation, selected Likert-scale response categories were collapsed into broader categories (e.g., “Agree” and “Strongly Agree”; “High” and “Very High”) to improve readability. Three outcomes were designated *a priori* as primary: family physician-initiated counseling on reproductive plans (Q7), age-related fertility decline (Q8), and oocyte cryopreservation (Q16). The study was designed as a descriptive survey based on a limited set of prespecified comparisons. Therefore, no multivariable modeling was undertaken, and no formal correction for multiple comparisons was applied. All subgroup comparisons other than the primary outcomes are considered exploratory and hypothesis-generating, with interpretation emphasizing the direction and magnitude of associations rather than nominal significance alone. However, all reported *p*-values reflect statistical comparisons across the complete, uncollapsed response distributions.

## 3 Results

### 3.1 Sample characteristics

A total of 401 family physicians completed the survey. The median age was 33 years (IQR: 29–37), and the sex distribution was nearly equal (49.4% female and 50.6% male). Regarding marital status, 44.4% were single, 51.4% were married, and 4.2% were divorced or widowed. The majority (56.9%) reported not having children. The median duration of clinical practice was 8 years (IQR: 5–10). Demographic characteristics are summarized in Table 1. Responses originated from 45 of Turkey’s 81 provinces and were concentrated in the Central Anatolia and Black Sea regions, with the single most frequent province accounting for 30.3% of responses.

**Table 1:** Demographic characteristics of study participants.

Characteristics	Overall sample (*n* = 401)
Age, years, median (IQR)	33 (29–37)
Sex	
Female	198 (49.4%)
Male	203 (50.6%)
Marital status	
Single	178 (44.4%)
Married	206 (51.4%)
Divorced/Widowed	17 (4.2%)
Parental status	
Has children	173 (43.1%)
No children	228 (56.9%)
Duration of clinical practice, years, median (IQR)	8 (5–10)

IQR, interquartile range (25th–75th percentile).

### 3.2 Fertility awareness counseling attitudes

Overall, 34.2% of respondents considered it appropriate for family physicians to proactively initiate discussions about patients’ reproductive plans, and 53.6% supported proactive discussion of age-related fertility decline. Among those who endorsed these conversations, the median recommended age to begin discussing reproductive plans was 30 years (IQR: 25–30), and for fertility decline, it was 35 years (IQR: 34–35). The majority of respondents (60.4%) considered that age-related fertility decline should be incorporated into routine gynecological follow-up or periodic health examinations.

Among those who opposed routine discussion, the most frequently cited reason was that the topic falls outside the family physician’s primary professional responsibility (28.4%), followed by insufficient consultation time (16.7%) and respect for patient reproductive autonomy (14.0%). Concerns about appearing to pressure patients to have children (7.0%), inducing patient anxiety (4.0%), and the belief that frequent discussion is unnecessary (1.5%) were cited less often.

Significant subgroup differences were observed across several items. Married physicians were more likely than their unmarried peers to endorse family physician-initiated discussion of both reproductive plans (42.2% vs. 25.6%, *p* < 0.001) and fertility decline (63.1% vs. 43.6%, *p* < 0.001). They recommended initiating these conversations at a younger age for reproductive plans (median 26 vs. 30 years, *p* < 0.001). Physicians with children showed a similar and consistent pattern relative to those without children (reproductive plans: 46.8% vs. 24.6%, *p* < 0.001; fertility decline: 65.3% vs. 44.7%, *p* < 0.001). Physicians aged ≥35 years were less likely to support routine inclusion of this topic in health visits (51.4% vs. 65.5%, *p* = 0.005) and more frequently cited respect for patient autonomy as a reason for not raising the topic (21.9% vs. 9.4%, p < 0.001). Physicians with ≥10 years of practice showed the same pattern: lower support for routine discussion (51.8% vs. 64.8%, *p* = 0.012) and more frequent citation of patient autonomy (22.6% vs. 9.5%, *p* < 0.001). Among sex-based comparisons, the only item reaching significance was a higher rate of male physicians reporting that fertility counseling falls outside their professional scope (33.5% vs. 23.2%, *p* = 0.023). Detailed subgroup comparisons are presented in Table 2.

**Table 2:** Fertility awareness counseling attitudes by demographic characteristics.

Variables	Total (*n* = 401)	Age, years			Sex			Marital status			Parental status			Practice duration, years		
		<35 (*n* = 255)	≥35 (*n* = 146)	*p*	Female (*n* = 198)	Male (*n* = 203)	*p*	Married (*n* = 206)	Not married (*n* = 195)	*p*	Children (*n* = 173)	No children (*n* = 228)	*p*	<10 (*n* = 264)	≥10 (*n* = 137)	*p*
Proactive counseling attitudes																
Q7. FP should initiate discussion on patients’ reproductive plans																
% Yes	34.2	31.8	38.4	0.181	36.4	32.0	0.359	42.2	25.6	**<0.001**	46.8	24.6	**<0.001**	30.7	40.9	**0.041**
Recommended age to initiate																
Median (IQR)	30 (25–30)	30 30–33)	25 (20–30)	**<0.001**	30 (25–30)	30 (25–33)	0.343	26 (23–30)	30 (30–35)	**<0.001**	27 (23–30)	30 (30–35)	**<0.001**	30 (30–33)	25 (20–28)	**<0.001**
Q8. FP should initiate discussion on age-related fertility decline																
% Yes	53.6	51.8	56.9	0.326	50.5	56.7	0.217	63.1	4t3.6	**<0.001**	65.3	44.7	**<0.001**	51.9	56.9	0.337
Recommended age to initiate																
Median (IQR)	35 (34–35)	35 (35–36)	35 (30–35)	**0.006**	35 (30–35)	35 (35–38)	**0.006**	35 (30–35)	35 (35–38)	**0.031**	35 (30–35)	35 (35–37)	**0.025**	35 (35–36)	35 (30–35)	**0.002**
Q9. Fertility decline should be part of routine health visits																
% Yes	60.4	65.5	51.4	**0.005**	64.7	56.2	0.082	48.5	72.8	**<0.001**	49.7	68.4	**<0.001**	64.8	51.8	**0.012**
Q10. Reasons for not addressing the topic during routine visits, %†																
Not within primary professional responsibility																
%	28.4	29.4	26.7	0.564	23.2	33.5	**0.023**	33.5	23.1	**0.021**	29.5	27.6	0.684	29.2	27.0	0.649
Insufficient consultation time																
%	16.7	18.0	14.4	0.421	14.1	19.2	0.174	20.9	12.3	**0.030**	17.9	15.8	0.571	18.9	12.4	0.128
Respect for patient reproductive autonomy																
%	14.0	9.4	21.9	**<0.001**	16.2	11.8	0.267	19.9	7.7	**<0.001**	20.2	9.2	**0.003**	9.5	22.6	**<0.001**
Concern about inducing patient anxiety or distress																
%	4.0	3.9	4.1	1.000	5.6	2.5	0.185	5.8	2.1	0.094	6.4	2.2	0.064	3.4	5.1	0.578
Concern about appearing to pressure patients to have children																
%	7.0	5.9	8.9	0.348	8.6	5.4	0.295	10.7	3.1	**0.005**	11.6	3.5	**0.003**	6.1	8.8	0.424
Raising the topic at regular intervals is unnecessary																
%	1.5	0.4	3.4	**0.026**	2.0	1.0	0.444	2.4	0.5	0.216	3.5	0.0	**0.006**	0.8	2.9	0.187

Data are expressed as % for binary items and median (IQR) for continuous items. Likert scale responses have been collapsed for display (e.g., “Agree” + “Strongly Agree”); all *p*-values reflect comparisons across complete, uncollapsed response distributions. Statistically significant *p*-values (*p* < 0.05) are shown in bold red. †Multiple choice item; respondents could select more than one option; percentages are of all participants. FP, family physician; IQR, interquartile range. Bold values indicate statistically significant p-values (*p* < 0.05).

### 3.3 Knowledge assessment

Regarding the age at which slight fertility decline begins, 60.6% of respondents selected the 30–34-year range, which is most consistent with current evidence, 30.9% selected the 35–39-year range, and 5.7% selected the 25–29-year range. For the onset of marked fertility decline, 54.1% selected 35–39 years, 37.9% selected 40–44 years, and 7.7% selected 45 years or older. For IVF live birth probability in women younger than 35 years, 51.6% selected the 30–39% range; 32.2% estimated 20–29, and 14.7% selected ≥40%, indicating overestimation in a notable subset. For women older than 35 years, 50.4% selected 20–29 and 38.9% selected 0–19%, while 10.5% selected 30–39%, suggesting a tendency toward overestimation in a subset of respondents.

Knowledge responses differed significantly by age and years of practice across most items, but not by sex. Physicians younger than 35 years were more likely to select the evidence-consistent range for both slight fertility decline (67.1% vs. 49.3%, *p* < 0.001) and marked fertility decline (62.0% vs. 40.4%, p < 0.001) compared with older peers. A parallel gradient was observed by practice duration: physicians with fewer than 10 years were more likely to select the correct range for slight decline (65.5% vs. 51.1%, *p* < 0.001) and marked decline (61.0% vs. 40.9%, *p* < 0.001). Interestingly, physicians with children and married physicians showed lower rates of evidence-consistent responses for the fertility decline items, mirroring the pattern observed for counseling attitudes. Sex was not significantly associated with any knowledge item. Full group-level distributions are presented in Table 3.

**Table 3:** Knowledge assessment responses by demographic characteristics.

Variables	Total (*n* = 401)	Age, years			Sex			Marital status			Parental status			Practice duration, years		*p*
		<35 (*n* = 255)	≥35 (*n* = 146)	*p*	Female (*n* = 198)	Male (*n* = 203)	*p*	Married (*n* = 206)	Not married (*n* = 195)	*p*	Children (*n* = 173)	No children (*n* = 228)	*p*	<10 (*n* = 264)	≥10 (*n* = 137)	
Reproductive biology knowledge																
Q11. Onset of slight fertility decline, % selecting 30–34 years†																
	60.6	67.1	49.3	**<0.001**	59.1	62.1	0.736	57.8	63.6	0.057	53.2	66.2	**<0.001**	65.5	51.1	**<0.001**
Q12. Onset of marked fertility decline, % selecting 35–39 years†																
	54.1	62.0	40.4	**<0.001**	52.5	55.7	0.581	47.6	61.0	**<0.001**	44.5	61.4	**<0.001**	61.0	40.9	**<0.001**
IVF outcome knowledge																
Q13. Live birth rate per IVF cycle in women <35 years, % selecting 30–39%†																
	51.6	51.8	51.4	**0.001**	50.5	52.7	0.141	48.5	54.9	0.100	48.6	54.0	**0.003**	51.1	52.6	**<0.001**
Q14. Live birth rate per IVF cycle in women >35 years, % selecting 0–19%†																
	38.9	42.4	32.9	**0.004**	35.9	41.9	0.173	36.9	41.0	**0.003**	35.3	41.7	**<0.001**	42.4	32.1	**0.001**

Percentages represent the response category most consistent with current evidence: 30–34 years for Q11, 35–39 years for Q12, 30–39% for Q13, and 0–19% for Q14 (see text for full distribution of Q13 and Q14). All p-values reflect comparisons across complete response distributions; the Fisher–Freeman–Halton test was used where applicable. Statistically significant *p*-values (*p* < 0.05) are shown in bold red. IVF, in vitro fertilization. Bold values indicate statistically significant p-values (*p* < 0.05).

### 3.4 Oocyte cryopreservation attitudes, scenario-based likelihood, and policy views

One-third of respondents (33.2%) reported being familiar or knowledgeable about OCP, while 16.5% reported no familiarity at all. Overall, 62.6% of participants believed that family physicians should initiate OCP-related discussions with eligible patients.

In the four clinical vignettes, willingness to discuss or refer for OCP was substantially higher in oncology scenarios: 87.8% rated their likelihood as high or very high for a 25-year-old woman newly diagnosed with cancer, and 88.0% for a 35-year-old counterpart. For social indications, willingness was markedly lower and age-dependent: only 32.7% rated their likelihood as high or very high for a 25-year-old deferring pregnancy for career or educational reasons, compared with 59.1% for a 35-year-old in the same scenario.

Regarding policy attitudes, 54.4% agreed or strongly agreed that the legal framework should support elective OCP for social indications, and 72.3% expressed support or strong support for insurance reimbursement. If OCP were fully covered by public health insurance, 57.9% anticipated that their likelihood of discussing the procedure would increase substantially or definitively. If elective OCP were legally recognized as a standard preventive health right, 72.6% reported their discussion frequency would increase.

Across virtually all OCP-related items, physicians younger than 35 years, those without children, unmarried physicians, and those with fewer than 10 years of practice reported significantly higher familiarity, stronger support for family physician-initiated OCP counseling, and greater endorsement of legal and insurance reforms (all *p* < 0.001 except where noted). Female physicians were significantly more likely than male physicians to support family physician-initiated OCP discussion (69.7% vs. 55.7%, *p* = 0.004), to agree that the legal framework should support social OCP (60.1% vs. 48.8%, *p* = 0.036), and were more often categorized as ‘somewhat familiar’, whereas males more often reported ‘not at all familiar’ (*p* = 0.003). Sex was not significantly associated with insurance coverage support, scenario-specific likelihood, or anticipated behavioral change. Detailed subgroup comparisons are presented in Table 4.

**Table 4:** Oocyte cryopreservation attitudes and policy views by demographic characteristics.

Variables	Total (*n* = 401)	Age, years			Sex			Marital status			Parental status			Practice duration, years		
		<35 (*n* = 255)	≥35 (*n* = 146)	*p*	Female (*n* = 198)	Male (*n* = 203)	*p*	Married (*n* = 206)	Not married (*n* = 195)	*p*	Children (*n* = 173)	No children (*n* = 228)	*p*	<10 (*n* = 264)	≥10 (*n* = 137)	*p*
Familiarity and general counseling attitudes																
Q15. Familiarity with OCP, % Familiar or Knowledgeable																
	33.2	42.4	17.1	**<0.001**	37.4	29.1	0.003	17.0	50.3	**<0.001**	11.6	49.6	**<0.001**	42.4	15.3	**<0.001**
Q16. FP should initiate OCP discussion with eligible patients																
% Yes	62.6	72.2	45.9	**<0.001**	69.7	55.7	0.004	48.5	77.4	**<0.001**	48.0	73.7	**<0.001**	71.2	46.0	**<0.001**
Q17. Likelihood of discussing or referring for OCP, % High or Very High																
Scenario A: 25-year-old woman with a new cancer diagnosis																
	87.8	94.5	76.0	**<0.001**	87.9	87.7	0.273	80.1	95.9	**<0.001**	75.1	97.4	**<0.001**	95.5	73.0	**<0.001**
Scenario B: 35-year-old woman with a new cancer diagnosis																
	88.0	93.3	78.8	**<0.001**	86.4	89.7	0.225	81.6	94.9	**<0.001**	77.5	96.1	**<0.001**	93.9	76.6	**<0.001**
Scenario C: 25-year-old woman deferring pregnancy for social reasons																
	32.7	38.4	22.6	**<0.001**	35.9	29.6	0.376	25.7	40.0	**<0.001**	24.9	38.6	**<0.001**	36.4	25.5	**<0.001**
Scenario D: 35-year-old woman deferring pregnancy for social reasons																
	59.1	67.1	45.2	**<0.001**	63.1	55.2	0.131	50.5	68.2	**<0.001**	48.6	67.1	**<0.001**	66.7	44.5	**<0.001**
Policy attitudes and behavioral intentions																
Q18. Legal support for elective OCP for social indications, % Agree or Strongly Agree																
	54.4	63.1	39.0	**<0.001**	60.1	48.8	0.036	41.7	67.7	**<0.001**	39.3	65.8	**<0.001**	64.0	35.8	**<0.001**
Q19. Insurance coverage support for OCP with social indications, % Support or Strongly Support																
	72.3	81.2	56.8	**<0.001**	71.7	72.9	0.596	58.7	86.7	**<0.001**	58.4	82.9	**<0.001**	81.4	54.7	**<0.001**
Q20. If OCP fully covered by insurance, counseling likelihood: % increase substantially or definitely																
	57.9	65.1	45.2	**<0.001**	60.6	55.2	0.150	47.1	69.2	**<0.001**	45.1	67.5	**<0.001**	67.0	40.1	**<0.001**
Q21. If OCP legally recognized as a preventive right, discussion frequency: % Yes, would increase																
	72.6	82.8	54.8	**<0.001**	71.2	73.9	0.822	59.2	86.7	**<0.001**	54.3	86.4	**<0.001**	83.3	51.8	**<0.001**

Familiarity: % “Familiar/Knowledgeable.” Scenario likelihood: % “High” or “Very High.” Legal support (Q18): % “Agree” or “Strongly Agree.” Insurance support (Q19): % “Support” or “Strongly Support.” Behavioral change if free (Q20): % “Would increase substantially” or “Would definitely increase”. Behavioral change if legalized (Q21): % “Yes, would increase”. All *p*-values reflect comparisons across complete response distributions. Statistically significant *p*-values (*p* < 0.05) are shown in bold red. OCP, oocyte cryopreservation; FP, family physician. Bold values indicate statistically significant p-values (*p* < 0.05).

## 4 Discussion

Our study shows that family physicians in Turkey are not entirely opposed to fertility counseling, but they do not yet appear to regard this area as an established part of routine primary care practice. While the proportion of those supporting proactive reproductive planning consultations remained at 34.2%, support for addressing age-related fertility decline rose to 53.6%, and support for raising the topic of OCP with appropriate patients increased to 62.6%. This distribution suggests that support for counseling may vary depending on the extent to which the topic is viewed by family physicians as part of routine care. However, positive attitudes were not consistently accompanied by adequate knowledge, as the rates of correct responses regarding age-related fertility decline and IVF success remained limited. Subgroup findings reveal that patterns regarding general fertility counseling and OCP do not align, and that the issue cannot be explained solely by a lack of information.

Reservations regarding proactive discussions about reproductive planning point to a multifaceted picture that cannot be reduced to a single cause. The limitations of primary care practices and the understanding of physicians about their roles as family medicine specialists seem to be effective on the approach to fertility counseling and guidance. The fact that 28.4% of physicians who do not support routine counseling view this issue as falling outside their primary professional scope of responsibility, while 16.7% cite time constraints and 14.0% cite respect for patient autonomy as reasons, is consistent with previous studies reporting barriers related to role definition, prioritization, and perceptions of professional responsibility in the implementation of preconception care (18, 19). In particular, short consultation times, competing preventive care agendas, and a lack of tools to facilitate counseling make it difficult for this topic to become established in routine practice (18, 20). Data from the oncology context also report time pressure as one of the primary barriers to fertility preservation consultations, suggesting that this issue cannot be reduced to merely a local lack of implementation (21). These findings indicate a need for brief and practical tools to make the role of preconception care and fertility counseling in primary care more concrete. Patient-side factors, including limited fertility-preservation knowledge and psychological barriers, may further compound these physician-level barriers (22).

Our study found a marked divergence in support for two counseling topics within the same field. While 34.2% of respondents considered it appropriate to proactively discuss reproductive plans, 60.4% supported addressing age-related fertility decline during routine visits. This suggests that information on age-related fertility may be perceived by physicians as a more standardized, biomedical aspect of preventive care (12). However, when pregnancy intentions are questioned in primary care using appropriate language and context, they are found to be acceptable and beneficial to women (23)—making it difficult to explain this difference solely through concerns about intrusiveness. Indeed, previous studies indicate that while healthcare professionals acknowledge the importance of age-related fertility decline, they do not consistently incorporate this into routine counseling (12, 24). Therefore, the issue may lie less in the clinical value of the topic and more in the communication approach and the practical infrastructure for when, how, and in which patients this guidance is required.

The proportion of participants who correctly identified the age range at which a mild and significant decline in fertility begins remained at 60.6 and 54.1%, respectively. Responses regarding the probability of a live birth following IVF also indicated that knowledge gaps persisted, with a significant proportion of participants overestimating the likelihood of success. It has previously been reported that healthcare professionals frequently misjudge the timing of age-related fertility decline, the changes in hormonal characteristics, and the impacts of other factors on fertility (7, 8). In fact, even though knowledge levels may be relatively high in fields closer to reproductive health, the knowledge gap is largely sustained (25). Indeed, among obstetrics and gynecology residents, the success rate of assisted reproduction is frequently overestimated, and there exist significant knowledge gaps regarding fertility and treatment outcomes among physicians in all fields (26, 27). Therefore, the need for continued education and in-service training should not be limited to certain groups. Rather, it seems beneficial to incorporate fertility training to a greater extent during medical training and to repeat these education measures throughout professional life for those in primary care. Otherwise, counseling may place patient expectations and referral timing on an overly optimistic foundation.

In our study, while the percentage of those reporting familiarity with OCP remained at 33.2%, the percentage of those who believed they should initiate an OCP-related discussion with appropriate patients reached 62.6%. This discrepancy suggests that counseling support does not always go hand in hand with sufficient method-specific knowledge and preparation. In a survey conducted among obstetricians in the U. S., although a high percentage of respondents stated that elective fertility preservation should be discussed, those who provided frequent counseling were more limited. The authors found that time and lack of knowledge were reported as the primary barriers (28). Similarly, among obstetric residents, there is a discrepancy in the frequencies of supporting fertility preservation discussions and actual practice of counseling even though it appears that those with a more positive attitude are physicians with better fertility knowledge (29). Data from the field of oncology also indicate that a positive attitude toward fertility preservation does not automatically align with specific method knowledge and readiness for implementation (30). As such, although positive attitudes toward fertility counseling are beneficial, the attitude alone does not translate into clinical behavior. In addition to the gap between acceptance and readiness, it is also evident that having better levels of knowledge on this topic may improve the likelihood and quality of fertility counseling.

Scenario-based responses showed that family physicians’ decisions regarding counseling for OCP vary significantly depending on the type of indication. While the tendency to recommend a consultation or referral remained very high in oncological scenarios (87.8–88.0%), this rate was lower in scenarios involving pregnancy deferral for social/personal reasons. This distribution suggests that OCP is perceived by physicians as a more clearly defined, time-sensitive fertility preservation intervention in an oncological context, whereas in planned use, the decision is viewed as a more selective area requiring more detailed counseling (13, 15). Indeed, while planned OCP is an ethically acceptable option, uncertainties regarding success rates, long-term outcomes, and an appropriate counseling framework continue to warrant clinical caution (17). Interestingly, subgroup findings indicate that attitudes toward general fertility counseling and OCP do not follow the same demographic pattern. While higher support for discussing reproductive plans and age-related fertility decline was observed among married physicians with children, familiarity with and support for OCP counseling were at greater levels among younger, single, childless, and less-experienced physicians. As these characteristics were strongly intercorrelated in our sample, these associations are descriptive and cannot be interpreted as independent effects. As such, the personal life experiences and professional status of physicians also seem to have a strong impact on the nature of counseling. Findings in the literature regarding physician gender, age, and years of experience are not unidirectional (31–33). These subgroup associations are exploratory and should be confirmed in adequately powered, prespecified analyses.

Findings at the policy level indicate that reimbursement regulations and the legal framework play a decisive role in physicians’ approach to fertility counseling. High costs and lack of insurance coverage for fertility preservation services have been identified as major barriers limiting referral rates and service utilization (34). Higher reported access to fertility counseling and preservation services in systems with state-level coverage suggests that finances can influence clinical practice (35, 36). In Turkey, official health guidelines define OCP as a legal indication for women undergoing chemotherapy or radiation therapy, for those facing surgeries that threaten reproductive function, and for those with low ovarian reserve who have not yet given birth or have a family history of early menopause (37). Therefore, within the primary care context, it must be carefully considered that educational interventions alone may not be sufficient, and that policy and financial regulations can also influence counseling practices. However, it should also be kept in mind that while these items reflect reported behavioral intentions, they do not directly demonstrate actual changes in clinical behavior.

Among the study’s strengths are the focus on questions specific to primary care practice and the simultaneous evaluation of knowledge, attitudes, scenario-based counseling tendencies, and policy views within a single design. However, the cross-sectional design does not allow for the establishment of causality, and the sample, which was formed based on voluntary participation and accessibility, carries the risk of limited representativeness and selection bias. Because recruitment relied on a single online professional network, the geographic distribution was uneven and concentrated in a few provinces; the sample is therefore not nationally representative, and regional generalization should be made with caution. The examined demographic and professional characteristics were strongly interrelated—younger physicians were predominantly unmarried, childless, and less experienced—so the reported subgroup associations are unadjusted and potentially confounded and were not subjected to multivariable adjustment, consistent with the descriptive aim of the study. The item assessing IVF success in women older than 35 years used a single broad category and did not differentiate prognoses across the late thirties and early forties, limiting the precision of the assessment of age-specific IVF knowledge. Self-reported data may not fully reflect actual clinical behavior and may be prone to overreporting of socially more acceptable responses. The survey was developed specifically for this study, and thus, psychometric validation is lacking. Subgroup findings are exploratory in nature, and responses to policy scenarios reflect hypothetical intentions rather than actual behavior. Therefore, the findings should be interpreted as data that provide a descriptive overview of fertility counseling in the primary care setting in Turkey—with the possibility to generate hypotheses for further studies.

## 5 Conclusion

Our study shows that family physicians in Turkey do not consistently consider the topics covered in fertility counseling to be part of their routine responsibilities, and that counseling support and the accuracy of information require optimization. There may be a need for a more structured educational approach regarding age-related fertility decline, the realistic limits of IVF success, and the social and medical indications for OCP in primary care. Standard triggers for counseling that facilitate the initiation of patient–physician discussion could support this process. Guidelines clarifying which topics should be addressed in different situations, including age, intentions, or treatments, could contribute to a more consistent approach in primary care. A simple fertility preservation referral algorithm extending from the family physician to the relevant center could reduce the reliance of counseling on personal initiative and improve the clinical decision-making process.

## Data Availability

The raw data supporting the conclusions of this article will be made available by the authors, without undue reservation.
